# Preparation and Applications of Electrospun Nanofibers for Wearable Biosensors

**DOI:** 10.3390/bios12030177

**Published:** 2022-03-17

**Authors:** Tengzhou Xu, Guojing Ji, Hui Li, Jiaduo Li, Zhou Chen, Desire Emefa Awuye, Jie Huang

**Affiliations:** 1 School of Aviation Engineering, Nanjing Institute of Industry Technology, Nanjing 210046, China; 2018100938@niit.edu.cn (T.X.); 2004100333@niit.edu.cn (J.H.); 2 School of Mechanical and Power Engineering, Nanjing Tech University, Nanjing 211800, China; a13813547709@163.com (G.J.); l787228170@163.com (H.L.); li799478199@163.com (J.L.); 3 Department of Minerals and Materials Engineering, University of Mines and Technology, Tarkwa 03123, Ghana; deawuye@umat.edu.gh

**Keywords:** electrospun nanofibers, wearable biosensors, membrane, morphology

## Abstract

The emergence of nanotechnology has provided many new ideas and innovations in the field of biosensors. Electrospun nanofibers have many excellent properties such as high specific surface area, high porosity, low cost, high efficiency, and they can be combined with a variety of sensors. These remarkable features have a wide range of applications in the field of sensors such as monitoring air pollutants, highly sensitive pressure sensors, and biosensors for monitoring the pulse of the body. This paper summarizes the working principle and influencing factors of electrospinning nanofibers, and illustrates their applications in wearable biosensors.

## 1. Introduction

Wearable energy supply devices are indispensable emerging wearable electronic products. Current energy supply devices are based on rigid, bulky materials, making them difficult to wear [1]. With the continuous development of sensors, people have made a great breakthrough in the field of wearables. The wearable sensor can be skin and environmentally friendly and has very good characteristics. For example, it can be cleaned, folded, clipped, and does not change the performance of the sensor. Therefore, as the performance of wearable sensors continues to improve, the application field is also expanding. Currently, many sensors are used to detect human health and physical movement, but the sensitivity, resistance to harsh environment and mechanical properties are not very good [2].

With the development of nanotechnology, nanofiber membranes have become an important branch of the sensor field [3,4]. Nanofiber membranes are widely used in the sensor field. Electrospinning is a simple and reliable preparation technique for the preparation of nanoscale fiber membranes [5,6]. The fiber film produced by electrospinning has many excellent characteristics and can meet the needs of modern technology [7]. This paper reviews the preparation, characteristics, and application of electrospun nanofibers in wearable sensors.

## 2. Electrospinning

### 2.1. Working Principle and Method

**Figure 1:**
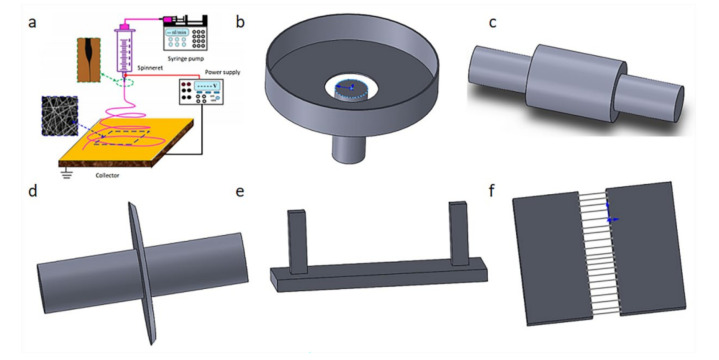
A typical electrospinning operation and different collector types. (**a**) Electrospinning principle diagram [8]; (**b**) water bath collector; (**c**) roller collector; (**d**) disc collector; (**e**) magnetized collector; (**f**) parallel plate electrode collector.

A nanofiber membrane is produced by the precursor solution under the dual influence of electrostatic force and surface tension. These forces tend to stretch and form a jet, causing the solute to accumulate on the receiving device. Figure 1 shows a typical electrospinning operation and different collector types. The electrospinning device mainly includes high voltage power supply, collector, and spinneret [8]. A high voltage electric field is applied between the collector and the spinneret, where the precursor solution is loaded. When the electrostatic force is greater than the surface tension, the solution is ejected from the Taylor cone at the tip. During this process, the solvent is volatilized and the solute is carried by the receiving device to form the fiber membrane. By modifying the electrospinning parameters, spinneret, and collector, many nanofiber films with different shapes and properties can be obtained [9].

### 2.2. Several Common Electrospinning Methods

Electrospinning technology has been developed for many years and has gradually advanced to date. During the development process, the electrospinning equipment was adjusted in order to obtain the nanofibers we wanted, for example, centrifugal electrospinning, coaxial electrospinning, multi-jet electrospinning, multilayer electrospinning, compound field electrospinning.

#### 2.2.1. Coaxial Electrospinning

**Figure 2:**
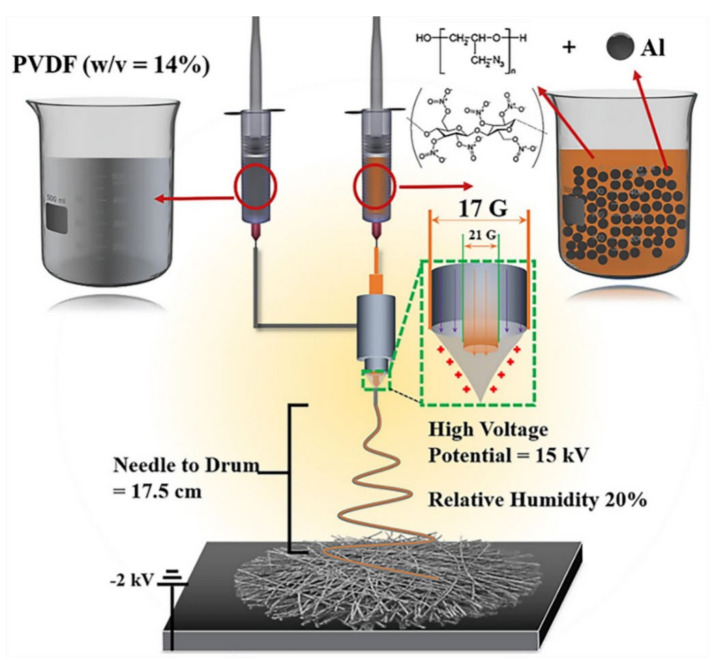
Schematic diagram of the preparation of the Al@GAP/NC/PVDF composite film by the coaxial spinning method [10].

The coaxial method is also a commonly used electrostatic spinning method. In this way, a nanofiber membrane with a core-shell structure is produced. The core-shell structure nanofiber membrane is more comprehensive than the single-structure nanofiber membrane. Compared with ordinary electrospinning, the coaxial method has an additional syringe pump and coaxial nozzle. Figure 2 shows the schematic diagram of preparation of the Al@GAP/NC/PVDF composite film by the coaxial spinning method [10]. It can be seen from the figure that two syringes are loaded with different solutions for spinning. The solution passes through the coaxial nozzle to form a core-shell structure. This structure can make the fiber more complex to improve performance. At the same time, it provides convenience for obtaining nanofibers with a hollow structure. This structure is usually used in situations where more types of solutions are required. 

#### 2.2.2. Centrifugal Electrospinning

**Figure 3:**
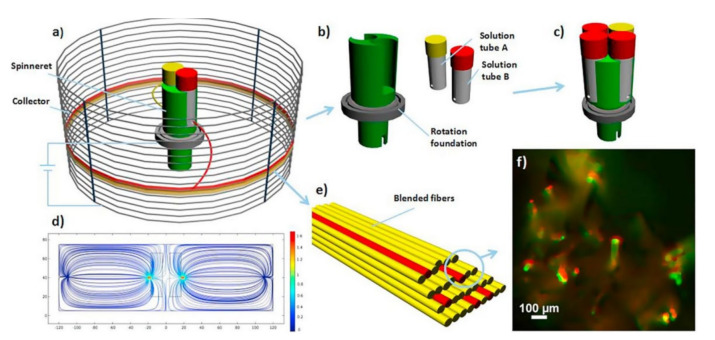
Schematic diagram of centrifugal electrospinning. (**a**) Structure chart; (**b**) rotating device; (**c**) nozzle is mounted on a rotating body; (**d**) electric field distribution map; (**e**) composite fiber; (**f**) fluorescence image of composite fiber [11].

Centrifugal electrospinning is also a widely used method at present. The major difference between centrifugal electrospinning and ordinary electrospinning is that the centrifugal method uses centrifugal force to stretch the solution to spin. The spinning in both ordinary electrospinning and coaxial electrospinning are controlled by an electric field force. The centrifugal electrospinning device is also mainly cylindrical. This method mainly relies on centrifugal and electrostatic forces to stretch the polymer solution. Figure 3 is a schematic diagram of centrifugal electrospinning [11]. The advantage of this method is that the spinning dope can be a solution or a polymer melt so that the applicable polymers are flexible and diverse.

#### 2.2.3. Multi-Jet Electrospinning

**Figure 4:**
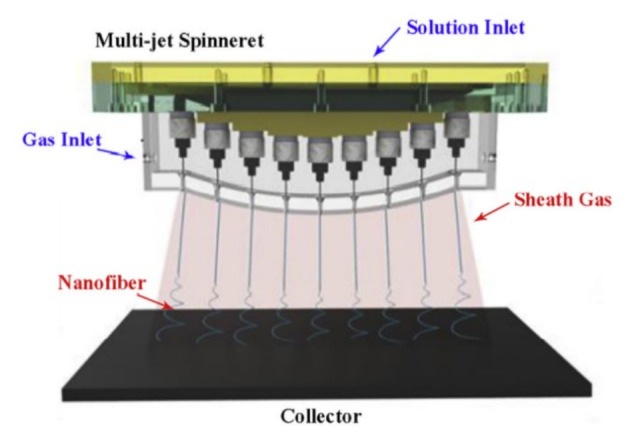
Schematic diagram of multi-jet electrospinning [12].

This method places different polymer solutions in different nozzles for spinning. The composite nanofibers obtained by multi-jet electrospinning can be combined and eventually improve the yield of electrospinning. Studies have shown that composite fiber materials have a wider application range than single polymer fiber materials. Figure 4 is a schematic diagram of multi-jet electrospinning [12]. Compared with ordinary electrospinning, it has more nozzles. There are no other significant differences apart from the number of nozzles. 

#### 2.2.4. Multilayer Electrospinning

**Figure 5:**
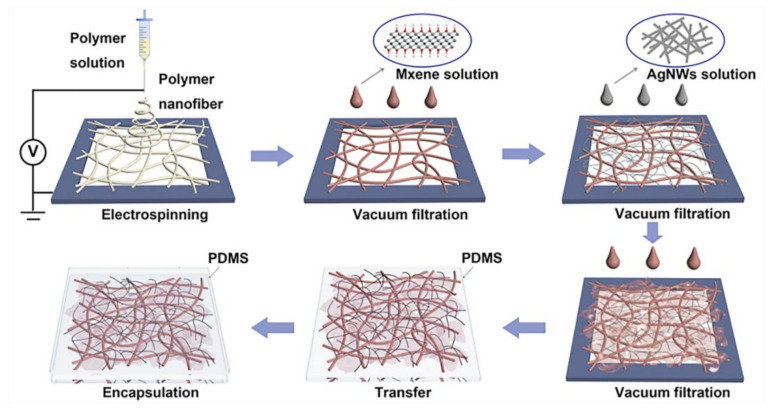
Schematic diagram of multilayer electrospinning [13].

Multi-layer electrospinning spins different polymer materials in sequence. These materials are deposited layer by layer on the collection device to form multiple layers of composite fibers. This composite fiber has a wide range of application. For example, when applied to artificial blood vessel stents, the pore size and porosity of the stent material are greatly increased. Figure 5 is a schematic diagram of multilayer electrospinning [13]. The difference between the multi-layer electrospinning and ordinary electrospinning is continuous spinning on the spun nanofiber membrane to form a multilayer structure.

#### 2.2.5. Compound Field Electrospinning

**Figure 6:**
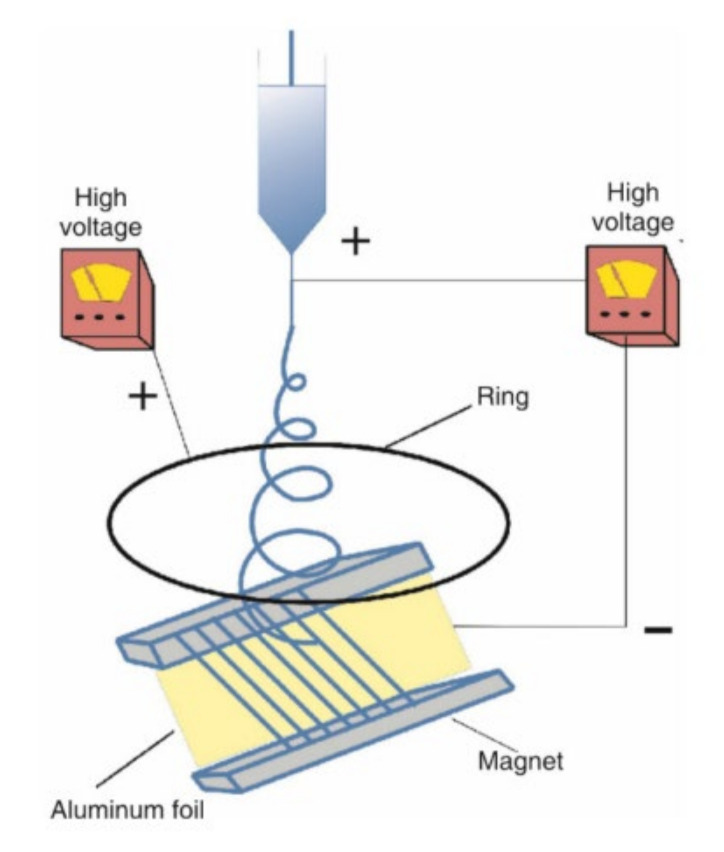
Schematic diagram of magnetic field electrospinning [14].

These fields are added to improve fiber morphology and performance or to make spinning smoother. For example, the purpose of increasing the ultrasonic vibration field is to reduce the viscosity of the polymer solution to make it easier to spin. This kind of compound field electrospinning is not much different from ordinary electrospinning and its working principle is basically the same. Figure 6 is a schematic diagram of electrospinning with increased magnetic field [14].

Several different electrospinning methods have been introduced as above. These are all modified on the basis of ordinary electrospinning equipment to obtain the fiber that is desired.

### 2.3. Impact Parameters

The formation of nanofibers is influenced by many factors such as solution concentration, voltage, flow rate, and the distance between the collector and the spinneret. In order to achieve the desired performance and appearance of the fiber film, the electrospinning parameters can be modified [15].

#### 2.3.1. Voltage

For traditional electrospinning, the increase in the voltage usually leads to a decrease in the fiber diameter, but if the voltage is too high, the droplets will be stretched unevenly and the solvent will not be able to volatilize, thereby increasing the fiber diameter. 

#### 2.3.2. Concentration

If the concentration of the precursor solution is too high, the entanglement of the molecular chain will be too much and the spinning will not proceed normally. However, if the concentration is too low, it is difficult to form a jet or the jet is easily broken during the drawing process to form bead fibers [16].

#### 2.3.3. Distance between the Collector and the Spinneret

As the distance decreases, the strength of the electric field increases, which leads to an increase in the stretching effect of the electric field force on the jet. This facilitates the formation of smaller diameter fibers but also shortens the draw time of the jet, resulting in insufficient solvent evaporation and non-uniform fibers. Conversely, a distance that is too large will cause the solvent to evaporate completely early and make it difficult for the fibers to deposit on the collector.

#### 2.3.4. Flow Rate

If the flow rate is too low, the instability of the Taylor cone will increase the instability of the jet and affect the morphology and structure of the fiber. However, if the flow rate is too high, the solvent will not volatilize enough, which will cause the fibers to bond and cause a lot of beading [17].

The above are several important parameters that affect electrospinning. The change in the parameters will directly affect the properties and morphology of the fiber membrane. As the core part of the sensor, the fiber membrane will directly affect the sensitivity and stability of the sensor and other important properties. For example, insufficient solvent evaporation can lead to beading. As a result, the loading capacity of the fiber is deteriorated and the sensitivity of the biosensor is reduced. Second, the increase in the fiber diameter will reduce the specific surface area and porosity of the fiber membrane, thereby reducing the sensitivity and lifetime of the biosensor.

### 2.4. Fiber Morphology

**Figure 7:**
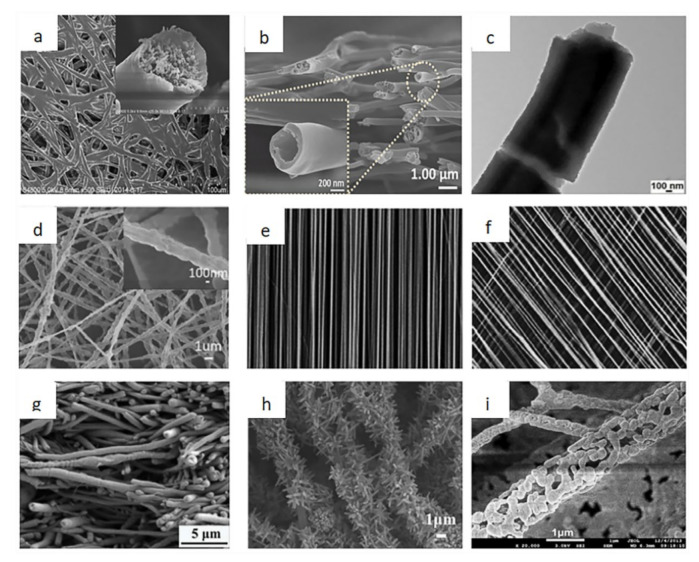
The different morphologies of the electrospun nanofibers. (**a**) Cobweb nanofiber [18]; (**b**) hollow nanofiber [19]; (**c**) core-shell nanofibers [20]; (**d**) randomly distributed nanofibers [21]; (**e**) aligned nanofibers [22]; (**f**) patterned nanofibers [23]; (**g**) composite nanofiber [24]; (**h**) pine needle nanofibers [25]; (**i**) hollowed-out nanofibers [26].

Different fibers can be obtained by changing the type of collector. This is as a result of different parameters such as machine structure and solution. Electrospun nanofibers will produce different morphologies such as core-shell structure, hollow structure, grid structure, cobweb structure, and so on. Figure 7 shows the different types of fiber morphologies [18,19,20, 21, 22, 23, 24, 25, 26].

## 3. Comparison of Electrospinning Wearable Sensors and Ordinary Sensors

### 3.1. How Electrospun Fibers Work in Sensors

The working principle of biosensors is mainly to react to the biological information of the human body through the added biological elements and convert them into electrical signals. For example, Kim et al. [27] created a transparent nanofiber hydrogel patch to detect glucose. The fibrous membrane works by adding glucose oxidase to react with glucose to give feedback. Young et al. [28] invented a wearable high-performance pressure sensor where the main material of the fiber membrane in this sensor was PVDF-HFP. Its working principle is to sense external pressure and convert it into an electrical signal, which is then fed back through the change of resistance. The high specific surface area of the electrospinning fibers allows these biological elements to fully contact the biological information of the human body to improve sensitivity. Not only that, but the electrospun fiber membrane also helps to increase the lifespan of the sensor. 

### 3.2. Performance Comparison with Ordinary Sensors

Electrospinning nanofibers are widely used in wearable sensors due to their excellent properties. Table 1 shows the performance comparison between the electrospinning sensor and other sensors.

It can be seen from the table that the electrospinning sensor has an absolute advantage in most performances. This is because the electrospun fiber membrane has the advantages of large specific surface area and high porosity. This also explains why electrospinning technology is widely used in the field of sensors. However, most of the solvents currently used have certain toxicity. The development of green solvent or even solvent-free methods is particularly important. It is believed that nanofiber sensors with higher sensitivity, longer life, and are more environmentally friendly can be developed through electrospinning technology in the future.

## 4. Electrospun Fibers for Wearable Sensors

As can be seen from the above, the electrospinning collector and the wide variety of fibers made have greatly increased its field application [37]. Wearable sensors have developed rapidly in recent years and electrospinning is widely used in the field of wearable sensors because of its simple operation and the many advantages of the fibers. Nanomaterials made by electrospinning technology have made a significant contribution to the development of wearable sensors. Nanomaterials have also developed from zero-dimensional nanoparticles to one-dimensional nanofibers and then to two-dimensional nanosheets [38]. Nanofibers are widely used in various fields due to their excellent properties. Among them, it is also widely used in the field of wearable sensors. Wearable sensors are mainly used to detect physical health and are mainly classified as pressure sensors, biological sensors, strain sensors, temperature sensors, and photoelectric sensors.

### 4.1. Wearable Pressure Sensor

**Figure 8:**
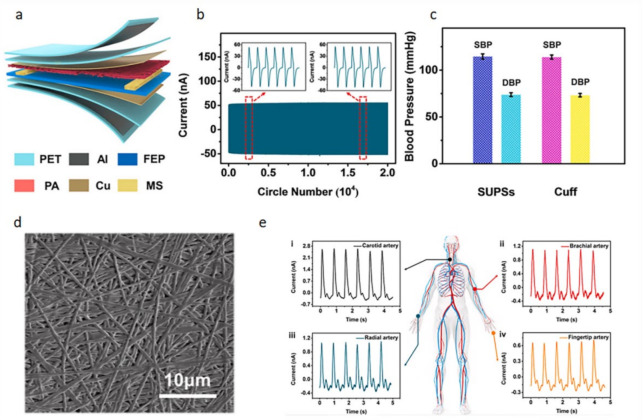
The construction diagram of the sensor and the monitoring diagram of blood pressure and pulse. (**a**) Schematic diagram of the sensor structure; (**b**) cycling stability of the sensor; (**c**) average blood pressure values measured by sensors and electronic sphygmomanometers; (**d**) SEM image of PA nanofiber membrane; (**e**) pulse signal under different arteries [41].

Pressure sensors mainly rely on pressure changes to produce feedback. The wearable pressure sensor can feedback the health of the body through the heartbeat or pulse [39]. This type of sensor can be placed on the chest, neck, wrist, and fingers [40]. The display is connected on the other end to observe the heart rate fluctuations. With the development of technology, such sensors are generally connected to smart watches. Xu et al. [41] invented a self-powered ultrasensitive wearable sensor for cardiovascular and pulse detection. See Figure 8, the sensor uses PA films and FEP films as triboelectric layers and copper foils as electrodes. The MS is assembled as a spacer between the triboelectric layers. Aluminum foil is attached to both sides of the sensor as an electrostatic shield. The FET film is assembled as an insulating layer between the electrodes and the electrostatic shield. Finally, the entire sensor is encapsulated by PET film. The PA film is subjected to cold pressing at 10 MPa after electrospinning. The pressure will drop rapidly after 30 s. It can be clearly seen that the fibers from cold-pressed electrospun PA film cross-linked together and had good morphology. It could also obtain higher electrical output signal as a triboelectric layer. Through the synergy of materials and devices, excellent sensing properties such as high sensitivity (10.29 nA/kPa), short response time (30 ms), and low detection limit (5 mg) are obtained. At the same time, the sensor can be measured in multiple parts of the body and has excellent stability.

On the other hand, a wearable pressure sensor can be placed on the finger to sense subtle pressure changes. The pressure can be accurately measured to the greatest extent. Lee et al. [42] invented a nano-mesh pressure sensor without sensory interference. The bottom and top of this sensor were layers of Au nanomesh. In the middle was a polyurethane nano-mesh layer coated with parylene and a polyurethane nano-mesh layer was introduced in the outermost layer. The diameter of the electrospun nanofibers was 200–400 nm. This structure ensures the mechanical durability and thinness of the sensor and also has high sensitivity both before and after friction (around 0.07 kPa^−^^1^).

**Figure 9:**
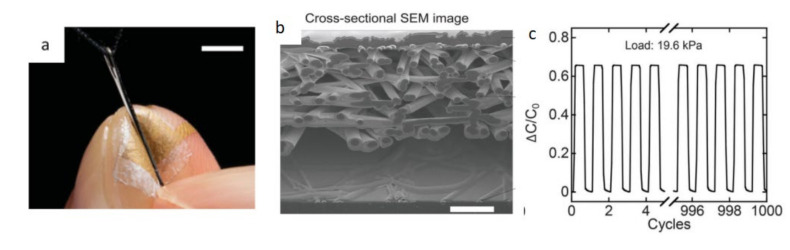
Schematic diagram of a pressure sensor without sensory interference. (**a**) Pressure sensor placed on index finger; (**b**) SEM image of the cross-section of the nanomesh sensor; (**c**) pressure sensitivity at 1000 cycles of pressure [42].

Figure 9 is a schematic diagram of a pressure sensor without sensory interference.

The current research directions are constantly improving the sensitivity, mechanical properties, electrical conductivity, and toughness of the sensors. That notwithstanding, simplifying the operation steps and reducing production costs as much as possible are also important issues. For wearable devices, microwave power transmission may be of great research value because it does not rely on wired power or batteries. Therefore, microwave power transmission is more stable and environmentally friendly.

### 4.2. Wearable Temperature Sensor

Most diseases the human body encounters causes a change in body temperature. The traditional method is to use an adhesive temperature sensor or infrared digital camera. Although these methods have some advantages, they cannot achieve economical and efficient continuous temperature monitoring [43]. For example, infrared cameras with high resolution and precision are expensive. The body’s normal temperature does not vary much, but abnormal and irregular temperature changes are considered signs of certain diseases. Therefore, it is a general trend to develop wearable sensors that can monitor changes in body temperature. 

**Figure 10:**
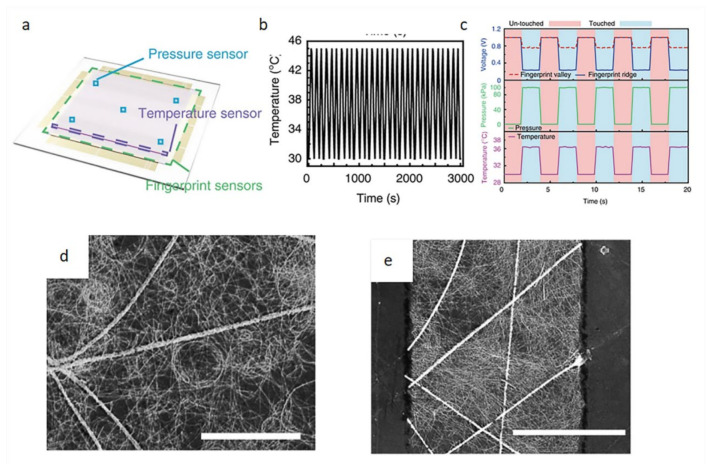
Sensor schematic diagram and performance characterization diagram. (**a**) Sensor schematic diagram; (**b**) stability graph with repeated loading and unloading; (**c**) sensor real-time sensorgram; (**d**,**e**) SEM images of hybrid electrode and patterned hybrid electrode [45].

In recent years, studies have shown that sensors that can monitor changes in body temperature must have high sensitivity and mechanical stability. Because the wearable sensor will move with the movement of the body, this may cause the performance of the sensor to decrease [44]. Wan et al. [45] invented a transparent and flexible fingerprint sensor that could be used to detect tactile pressure and skin temperature, see Figure 10. This sensor is a composite of a pressure sensor, temperature sensor, and fingerprint sensor. It is a continuous network of Ag nanofibers formed by electrospinning and the AgNWs are adhered to the surface of the fibers by electrospraying. It can clearly be seen that the AgNWs successfully adhered to the fiber surface without damage. The fiber diameter was 338 ± 35 nm. AgNWs were 30 ± 5 nm in diameter and 25 ± 5 mm in length. The sensing capability of the capacitance change was 17 times higher than that of traditional ITO electrode sensors. This sensor can have good temperature sensing up to 3000 cycles.

Various studies have shown that the development of temperature sensors tends to be more convenient and versatile. Therefore, the selection of raw materials will tend to provide materials with good degradability and biocompatibility. The operation of electrospinning is simple and the configuration of raw materials is relatively simple. Therefore, electrospinning is a good choice for this type of wearable sensor [46].

### 4.3. Wearable Biosensor

The development of wearable bioelectronics technology is greatly expanding the horizons of personalized health monitoring [47]. Wearable biosensors are mainly used to monitor sweat, sleep, blood sugar, and blood pressure. These monitored parameters contain a wealth of biomarkers. This information provides a way to gain insights into human health [48]. The development of sensors is now becoming more convenient, aiming at receiving the most comprehensive data with the least user intervention [49]. 

**Figure 11:**
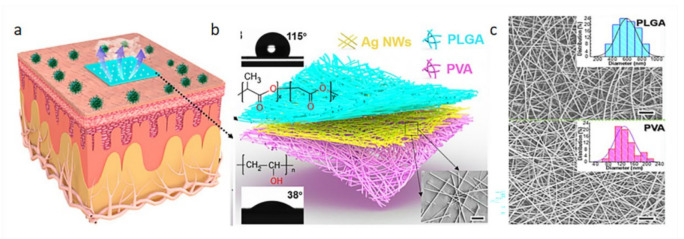
Schematic diagram of electronic skin and its scanning electron micrograph. (**a**) The electronic skin is attached to the epidermis; (**b**) electronic skin structure diagram; (**c**) SEM image of the upper and lower layers of material [51].

In recent years, the development of wearable biosensors has been very rapid. Its performance in areas such as sensitivity, mechanical properties, and biocompatibility are constantly improving. Jang et al. [50] invented a smart contact lens and transparent heat patch to treat chronic ocular surface inflammation. The feature of this sensor is that it can use a mobile phone to analyze the biological information in the wearer’s tears to achieve the purpose of timely diagnosis and automatic hyperthermia. Peng et al. [51] invented a new type of electronic skin. This electronic skin is flexible and biodegradable. It was the first to adhere AgNW on the surface of PVA fiber membrane by vacuum filtration. The PLGA solution was then electrospun directly onto the fiber membrane from the previous step. It can be seen from the figure that the nanofiber morphology of these two materials is excellent. This multi-layer staggered structure increases the specific surface area and porosity of the fiber membrane. Therefore, the sensor has a maximum matching peak power density of 130 mWm^−^^2^ and a voltage response sensitivity of 0.011 kPa. Figure 11 is a schematic diagram of the electronic skin and its scanning electron microscope image. Although this kind of electronic skin can be used for whole body monitoring, the degradability of its components needs to be improved. Additionally, this electronic skin may be affected by sweat or other pollutants.

At present, the development of wearable biosensors is mainly devoted to practicability and comprehensiveness. Moreover, the performance of wearable biosensors has been continuously improved with the development of electrospinning such as flexibility and affinity.

### 4.4. Wearable Strain Sensor

Human movement can also reflect human health. Therefore, monitoring human movement through sensors is also a reliable direction. Sensors can detect abnormal movements and sudden tremors. Such real-time surveillance is crucial for the early detection and treatment of disease [52]. Monitoring of human movement includes both major and minor movements such as walking, running, and swallowing. Various wearable sensors have been built to monitor these different types of motion [53,54,55].

**Figure 12:**
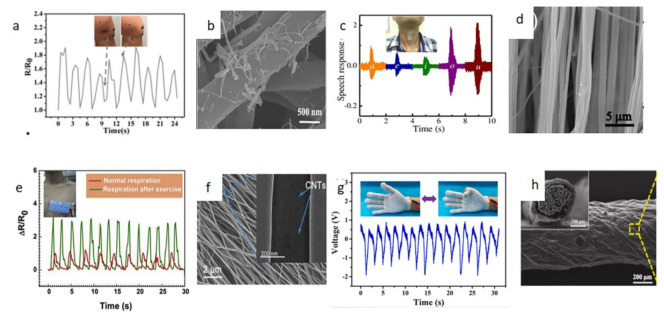
The response of the sensor in different parts and the SEM image of the fiber membrane. (**a**,**b**) knee bends [57]; (**c**,**d**) swallow [58]; (**e**,**f**) heart beats [59]; (**g**,**h**) Finger bends [60].

In recent years, electrospun wearable sensors have developed rapidly due to their excellent performance. However, improving its performance and reducing costs are still challenges to overcome [56]. Lin et al. [57] developed a flexible, multifunctional, wearable and conductive nanofiber composite material. The bottom layer of this sensor adheres to the modified CNTs on the surface of TPU nanofibers by ultrasonic vibration. The middle layer adsorbs AgNWs to the surface of the underlying fibers through the principle of capillary action. The outermost layer is wrapped with PDMS. It can be seen from the figure that the AgNWs were well adhered to the fiber surface. The synergistic effect of this multilayer structure improved the conductivity (3506.8 s/m) and sensitivity (1.36 × 10^5^). Figure 12 is a schematic diagram of human movement detection [57,58,59,60]. The tiny movements of the limbs can be reflected by sensors. The sensor is connected to a resistance meter, and the change in the resistance reflects the amplitude of the limb change.

Wearable strain sensors must have good flexibility to cope with the deformation caused by body movement. Moreover, the sensitivity of the sensor is generally measured by a resistance meter to test the resistance change. The more obvious the resistance change, the more sensitive the sensor.

### 4.5. Wearable Photoelectric Sensor

**Figure 13:**
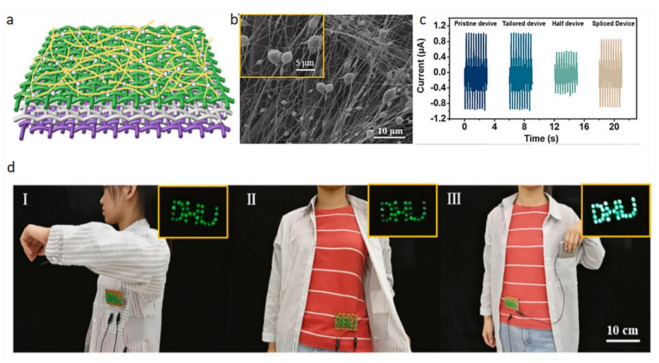
Model diagram and performance diagram of power generation fabric. (**a**) Model diagram, (**b**) SEM images, (**c**) voltage output under different conditions, (**d**) with different body movements, and the LED lights will glow [61].

Wearable energy supply equipment is a necessity of new wearable electronic products. Electrospinning, as a reliable and simple technology, provides a new research direction for this wearable energy supply equipment. Wearable sensors can obtain energy from human movement and effectively convert it into electrical energy. Qiu et al. [61] developed a highly flexible, breathable, tailorable, and washable power generation fabric for wearable electronic products. It is the simultaneous electrospinning and electrospraying that enables PVDF nanofibers and PTFE nanoparticles to adhere to the fiber surface. This practice increases the rough structure of the fiber surface and thus increases the specific surface area. The electrical output performance of the sensor is significantly improved through this synergistic effect. It can be seen from the figure that the nanofibers and nanoparticles remained structurally intact after washing and the voltage output was not affected by clipping. This sensor is capable of generating a power density of 80 mW/m^2^ at a load of 50 MΩ and the water vapor transmission rate reached 8.8 kgm^−2^ d^−1^. And the sensor can vary the brightness of the LED light depending on how much the arm swings. Figure 13 shows the model and conductivity of the power generation fabric. 

The performance of the power generation fabric is very stable. The amount of electricity generated varies with the amplitude of body movement. This kind of power-generating fabric can be well applied to clothing and make outstanding contributions to the development of smart clothing. Veeramuthu et al. [62] developed a durable smart clothing that provides a stable output current.

The rise in power generation fabrics provides a new direction for power equipment. Traditional power supply equipment is based on chemical batteries, which are bulky and inconvenient to replace [63]. The convenience and simplicity of wearable devices are unmatched by traditional chemical batteries. Moreover, the wearable fabric has good air permeability and novel colors. These characteristics greatly increase the application range of wearable sensors [64].

## 5. Conclusions

In recent years, the rapid development of electrospinning technology has brought new ways of thinking to all walks of life. Electrospinning technology is also widely used in the field of sensors. Electrospun nanofibers have many properties such as high porosity, large surface area, good flexibility, and so on. These remarkable performances have laid a solid foundation for the development of wearable sensors. Wearable sensors have attracted much attention as a relatively new research direction. The application range of wearable biosensors is also very broad. For example, it can monitor all aspects of the body’s movement to reflect the body’s health level. This article reviews the main mechanisms and influencing factors of electrospinning technology. It also explains their application in the field of wearable sensors. At the same time, it explains their application in the field of wearable sensors and exemplifies several specific application methods.

Although electrospinning technology has made some important breakthroughs in the field of wearable sensors, further in-depth research is needed in industrial production and normal human wear. With the development of electrospinning technology, various new types of nanofibers can be applied to sensory sensors to improve their performance.

## Figures and Tables

**Table 1:** Performance comparison with other sensors.

		Sensitivity	Linear Range	Stability	Ref.
Glucose Sensor	Electrospinning	4022 μAmM^−1^ cm^−2^	0.0002–1 mM	——	[29]
	others	91.8 μAmM^−1^ cm^−2^	0–13.0 mM	20 (90%) 100 (60%)	[30]
Blood pressure sensor	Electrospinning	60.28 kPa^−1^	0–24 kPa	13,000	[31]
	others	6.19 kPa^−2^	0–6 kPa	——	[32]
Body temperature sensor	Electrospinning	5.76 °C^−1^	24–48 °C	100	[33]
	others	8.962 nm°C^−1^	33–43 °C	——	[34]
Pressure Sensor	Electrospinning	1.49 kPa^−1^	——	around 1000	[35]
	others	0.24 kPa^−1^	10.5–96.25 kPa	around 1000	[36]

## Data Availability

Not applicable.
